# Investigating the relationship between physical cognitive tasks and a social cognitive task in a wild bird

**DOI:** 10.1007/s10071-024-01892-4

**Published:** 2024-07-26

**Authors:** Grace Blackburn, Benjamin J. Ashton, Alex Thornton, Holly Hunter, Sarah Woodiss-Field, Amanda R. Ridley

**Affiliations:** 1 Centre of Evolutionary Biology, School of Biological Sciences, University of Western Australia, Perth, WA Australia; 2 School of Natural Sciences, Macquarie University, Sydney, NSW Australia; 3 Centre for Ecology and Conservation, University of Exeter, Penryn, UK

**Keywords:** Cognition, General intelligence, Magpies, Social cognition, Wildlife

## Abstract

Despite considerable research into the structure of cognition in non-human animal species, there is still much debate as to whether animal cognition is organised as a series of discrete domains or an overarching general cognitive factor. In humans, the existence of general intelligence is widely accepted, but less work has been undertaken in animal psychometrics to address this question. The relatively few studies on non-primate animal species that do investigate the structure of cognition rarely include tasks assessing social cognition and focus instead on physical cognitive tasks. In this study, we tested 36 wild Western Australian magpies (*Gymnorhina tibicen dorsalis*) on a battery of three physical (associative learning, spatial memory, and numerical assessment) and one social (observational spatial memory) cognitive task, to investigate if cognition in this species fits a general cognitive factor model, or instead one of separate physical and social cognitive domains. A principal component analysis (PCA) identified two principal components with eigenvalues exceeding 1; a first component onto which all three physical tasks loaded strongly and positively, and a second component onto which only the social task (observational spatial memory) loaded strongly and positively. These findings provide tentative evidence for separate physical and social cognitive domains in this species, and highlight the importance of including tasks assessing both social and physical cognition in cognitive test batteries.

**Supplementary Information:**

The online version contains supplementary material available at 10.1007/s10071-024-01892-4.

## Introduction

Cognition, broadly defined as the ways in which individuals process, store, and act on environmental information (Shettleworth 2010), is vital for animal species, because it is involved in many aspects of life, from foraging, to competition, predator avoidance, and behavioural flexibility (Morand‑Ferron 2017; Lee and Thornton 2021; Szabo et al. 2022). Despite its importance, our understanding of how different cognitive traits have evolved, and the selection pressures behind the evolution of cognition in non-human animals (hereafter animals), continues to be a source of debate. One school of thought argues that selection pressures act on specific aspects of cognition that may be of particular importance to the ecology or life history of a species. For instance, group-living species may have more advanced social cognition compared to physical cognition (Humphrey 1976), while food caching species may have more advanced spatial memory abilities compared to other cognitive abilities (Krebs et al. 1989; Pravosudov and Roth 2013). In contrast, other researchers suggest that animal cognition is underpinned by a broad, general cognitive factor that underlies all cognitive domains, similar to that observed in humans (Burkart et al. 2017).

In humans, individual performance across different cognitive tasks is typically correlated, and multiple studies have described a general intelligence factor, *g*, that explains ~ 40% of variation in within-individual cognitive performance (Burkart et al. 2017). Similarly, studies across a number of primate species have found evidence to support the existence of *g* (Herndon et al. 1997; Banerjee et al. 2009; Reader et al. 2011). For example, a PCA on rhesus monkey (*Macaca mulatta*) individuals tested on 6 physical tasks found that *g* (i.e. the first PCA component) accounted for 48% of variation in cognitive performance (Herndon et al. 1997). Similarly, a study on captive cotton-top tamarin monkeys (*Saguinus oedipus*) supported the presence of *g*, with performance across 11 different tasks, including one social task, loading positively onto a single factor (Banerjee et al. 2009). Conversely, other studies have argued instead that cognition in primate species was divided into two distinct domains; (1) a physical domain, and (2) a social domain, and it is this division that inspired the development of the primate cognitive test battery (PCTB) (Tomasello and Call 1997; Herrmann et al. 2007). The physical domain comprises cognitive abilities related to navigating space, discriminating between quantities, and understanding causality, and is often thought to have evolved in the context of foraging (Hermann et al. 2007). Conversely, the social domain comprises abilities that are thought to have evolved due to the social challenges involved with group-living (namely cooperation and competition) and are those that deal with communication, social learning, and theory of mind (Hermann et al. 2007). Despite this initial separation of cognitive traits into distinct physical and social domains, Hermann et al. (2010), on re-examination of their 2007 data, found that cognition in chimpanzees was instead split into a spatial domain (consisting of tasks associated with tracking and locating a reward) and a physical-social domain (consisting of tasks associated with quantity discrimination, causality, and communication), while in young children, cognition was split into three cognitive domains (spatial, physical, and social). Despite over a decade of research into primate cognition since the development of the PCTB, whether there exists a general cognitive factor in animals analogous to *g* in humans (Deaner et al. 2006; Reader et al. 2011), or multiple distinct cognitive domains (Herrmann et al. 2010; Herrmann and Call 2012; Amici et al. 2012) is still an area of debate.

Beyond primates, studies investigating cognition in rodents, dogs, and birds using cognitive test batteries have generally reported evidence in support of a general cognitive factor akin to *g* (Galsworthy et al. 2005; Isden et al. 2013; Shaw et al. 2015; Arden and Adams 2016; Burkart et al. 2017; Ashton et al. 2018a; Soravia et al. 2022). This includes the study species for this research, the Western Australian magpie (*Gymnorhina tibicen dorsalis*) where a single component in PCA analysis accounted for 64.6% of the variation in cognitive performance across four physical tasks (Ashton et al. 2018a). However, test batteries presented to non-primate species tend not to include tasks assessing social cognition (Burkart et al. 2017; Shaw and Schmelz 2017), instead focusing solely on physical tasks, and often on tasks that may be underpinned by the same cognitive processes (van Horik and Lea 2017; Shaw and Schmelz 2017). For example, the majority of tasks presented in mice test batteries are thought to be underpinned by spatial cognition rather than multiple distinct cognitive domains (Herrmann and Call 2012; Amici et al. 2012; Shaw and Schmelz 2017). One study in a non-primate species that incorporated tasks assessing social cognition administered a PCTB-adapted battery of tasks to eight captive, hand-raised ravens (*Corvus corax)*, and found that ravens performed comparably in both physical and social tasks (Pika et al. 2020). However, this study did not employ standard statistical approaches such as PCA, Factor Analysis or Bayesian latent variable analysis to test for the presence of an underlying general intelligence factor. Therefore, whether social and physical cognition align in non-primate species remains undetermined, and further studies incorporating social cognitive tasks are needed.

In this study, we tested Western Australian magpies on a battery of four cognitive tasks representing cognitive traits of both the physical and social domain (associative learning, spatial memory, numerical assessment, and observational spatial memory respectively) to investigate whether cognition in this species aligns with a general factor, or multiple cognitive domains.

## Methods

### Study species and site

Data for this study were collected on an established population of Western Australian magpies located in Guildford and Crawley, Western Australia, between March and October 2022. Western Australian magpies are group-living passerines that live in year-round territorial groups and breed cooperatively (Pike et al. 2019). This subspecies is sexually dichromatic and lives in stable territorial groups (Ashton et al. 2018a). The study population consists of 16 groups of magpies (ranging in size from 2 to 12 individuals) that are habituated to people and individually identifiable via unique colour ring combinations (Ashton et al. 2018a).

### Cognitive test battery

Cognitive testing was conducted in the early morning between 5 and 11 a.m., when magpies are most active (Edwards et al. 2015). Tests were conducted when birds were in social isolation, with all other group members at least 10 m away, to minimise confounding effects of social learning, local enhancement, or interference (Ashton et al. 2018a). Adult magpies were selected for cognitive testing based on their sex and group (we aimed to test an even number of males and females, and to test birds across all focal groups in our study population). Cognitive tasks were presented successively to birds in a randomised order, with all tasks for a focal bird completed within 2 months.

**Fig. 1:**
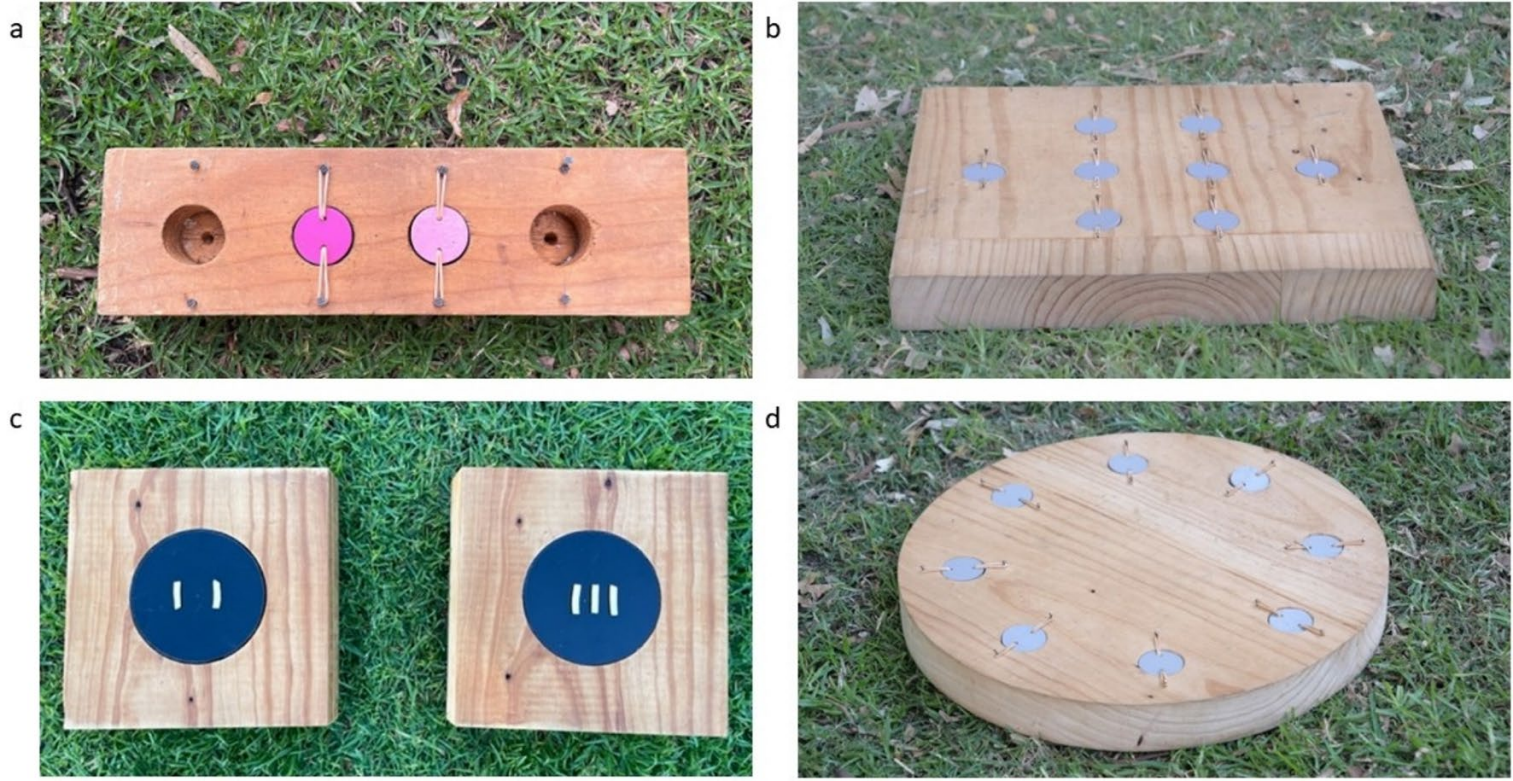
Cognitive tasks: **a** the associative learning task with the pink colour combination, **b** the spatial memory task, **c** the numerical assessment task with the 2 v 3 ratio combination, **d** the observational spatial memory task

We presented each individual with four cognitive tasks (Fig. 1). Tasks were designed to quantify a range of ecologically relevant cognitive traits:

- Associative learning is considered to be important for behaviours such as foraging, interactions with conspecifics, and predator avoidance, as it allows for the creation of predictive contingencies between cues in the environment (Richards 1979; Ward-Fear et al. 2016; Morand-Ferron 2017).
- Spatial memory is likely to be important in remembering the location of resources and territory boundaries (Sherry 1998; Maille and Schradin 2016; Ashton et al. 2018a).
- Numerical assessment is considered important for interactions with conspecifics and for foraging, as the ability to appropriately assess the number of competitors (Radford 2003), or the quantity of food in a foraging patch (Uller et al. 2003), may be highly beneficial for individuals (Benson‑Amram et al. 2017; Szabo et al. 2023).
- Observational spatial memory is an element of social cognition, thought to be important in food caching and group-living species, where observing and remembering the behaviour of others, particularly in a spatial context, is likely to benefit individuals (Bednekoff and Balda 1996; Brodin and Urhan 2014; Vetter et al. 2023).

The magpies tested in this study have participated in causally similar but visually distinct associative learning and spatial memory tasks previously (Ashton et al. 2018a; Blackburn et al. 2022), therefore individuals did not require training to complete these tasks. Previous work on this population found that repeated testing on causally identical but visually distinct inhibitory control tasks did not improve the cognitive performance of individuals (Sollis et al. 2023), suggesting that previous experience does not confound cognitive performance in this species if tasks are visually distinct. Small strands of mozzarella cheese were used as a food reward in all cognitive tasks.

### Associative learning

The associative learning task required individuals to learn an association between a colour shade and a food reward. The task consisted of a rectangular wooden block (31 × 9 × 4 cm) with four circular wells drilled into it, two of which were covered with plastic PVC lids painted different shades of the same colour (Fig. 1a). Prior to testing, the inside of each well was rubbed with mozzarella cheese, to prevent birds from using olfactory cues to locate the food reward. The PVC lids on wells were held in place by elastic bands and would swivel when pecked so birds could access the food reward beneath them. This task required magpies to peck down at the lids to access the food rewards in the wells, and thus resembled the natural foraging behaviour of magpies. For each individual, one colour shade was randomly chosen as the rewarded colour. Different shades of the same colour were used to avoid any potential confounding effects of colour preference (Rowe and Healy 2014; Ashton et al. 2018a; Osbrink et al. 2021; Connelly et al. 2022; Soravia et al. 2022).

Testing followed the protocol used by Shaw et al. (2015) and Ashton et al. (2018a, b), whereby individuals in their first trial were permitted to peck at and search both wells in order to determine that only one well contained a food reward. In all subsequent trials, birds were only allowed to peck at one well before the task was removed and were only permitted to eat the food reward if they pecked at the rewarded well first, so that there was a cost associated with pecking at the incorrect well. The task was randomly rotated to ensure birds were learning to associate the colour shade, and not the side of the array, with the food reward. Individuals were considered to have passed the task when they pecked the rewarded lid first in 10 out of 12 consecutive trials (Shaw et al. 2015; Ashton et al. 2018a). We quantified the associative learning score of individuals as the number of trials taken to reach this criterion (higher scores mean individuals took longer to learn the association and so indicate worse task performance).

### Spatial memory

To quantify spatial memory performance, we used a wooden foraging grid (36 cm x 40 cm × 4.5 cm) with eight wells drilled into it that were covered with light grey plastic PVC lids (Fig. 1b). The eight wells were arranged such that the first and third row contained two wells, and the middle row contained 4 well. Wells were rubbed with mozzarella cheese prior to testing to ensure birds were not using olfactory cues to locate the food reward. Testing followed the protocol used by Shaw et al. (2015) and Ashton et al. (2018a, b), but with one additional testing phase. For each individual, one well was randomly selected to be the rewarded well throughout all phases of the spatial memory task. Spatial memory testing consisted of six total phases within three stages: (1) the training stage, (2) the testing stage, (3) the probe stage. In the initial training stage of the experiment, birds were presented with the spatial memory task twice and allowed to search the foraging grid until they found the rewarded well and consumed the food reward. The foraging grid was then removed and rebaited, and after five minutes, this was repeated for the second phase of the experiment. The testing stage of the experiment consisted of a third, fourth, and fifth presentation of the foraging grid 24, 48 and 72 h after the initial training stage. The third and fourth presentations of the spatial memory task followed the protocol of Shaw et al. (2015) and Ashton et al. (2018a), and a fifth presentation was added as previous spatial memory testing in birds has found performance to significantly improve over time or number of trials (Sewall et al. 2013; Ashton et al. 2018a). Spatial memory performance was quantified as the combined total number of wells searched prior to finding the food reward (including re-visits to the same well) in the 24-, 48-, and 72-h presentations of the task (sensu Shaw et al. 2015, and Ashton et al. 2018a, b).

Following the training and testing stages, a sixth and final presentation of the spatial memory task was performed. This last presentation was a ‘probe’ trial, included to ensure individuals were not using olfactory cues to locate the food reward. For this trial, the spatial memory grid was rotated 180° (out of sight of the focal individual) and presented to the bird with no well rewarded. The foraging grid therefore appeared the same to the focal bird, but the previously rewarded well was now on the opposite side of the grid and was no longer rewarded. If the focal bird was using olfactory cues to locate the food reward, they would be expected to search this previously rewarded well that is now on the opposite side of the foraging grid. However, if birds were using spatial cues, they would be expected to search the well that is now in the rewarded position. The number of wells the focal bird searched before reaching the well that is now in the rewarded position was counted and considered to be their olfactory score, after which the board was removed by the experimenter. We then compared the focals bird’s olfactory score to their 72-h spatial memory score. If these scores were significantly different from each other, it would suggest that magpies are using olfactory cues to locate the food reward. If these scores were not significantly different from each other, it would suggest magpies are using spatial cues to locate the food reward.

### Numerical assessment

To quantify numerical assessment, we used two identical wooden boards (20 cm × 20 cm × 4.5 cm), each with a well (10 cm diameter, 3 cm deep) drilled into it that was covered with a black lid that was immovable to magpies (Fig. 1c). Wells were filled with mozzarella cheese to control for olfactory cues that may influence which board the magpie selected. Strands of mozzarella cheese were cut to 2.5 cm long and placed on the two black lids of the boards in four different ratios; 2 vs 2, 2 vs 3, 2 vs 4, and 2 vs 5. The black lids were marked identically with six small lines, 1 cm equidistant from each other, to mark where cheese strands would be placed and to make sure the horizontal space that the food reward was presented across remained consistent (i.e. the two strands of cheese would be placed between the 1st and 2nd, and 5th and 6th lines, while the three strands would be placed between the 1st and 2nd, 3rd and 4th, and 5th and 6th lines; Supplementary Material Figure S1). This was done to ensure birds were not selecting the board with food that took up the most amount of horizontal space and were rather selecting the board with the greater number of cheese strands. For each trial, cheese strands were placed on the black lids of the boards, and boards were placed equidistant in front of the focal bird, 1 m from the focal bird and 30 cm from each other. The experimenter then stepped back between 1-2 m from the boards [this was feasible because individuals in this study population are well habituated to the presence of experimenters (Pike et al. 2019; Blackburn et al. 2022)], allowing the bird to approach the boards and select the food reward from one board, which was considered the focal bird’s choice. Birds were allowed to consume the entirety of the food reward only on the board that they first chose, and following this both boards were removed.

Each of the four ratios was presented to focal birds once a day for 15 days, resulting in a total of 60 trials per bird. The four ratios were presented in a randomised order, and the side holding the greater number of cheese strands was alternated at random to ensure birds were selecting an amount of food, and not a side. We quantified an individual’s numerical assessment score as the number of times they selected the smaller amount of food in the 60 trials, such that a higher numerical assessment score was indicative of worse performance, in line with other tasks.

### Observational spatial memory

The observational spatial memory task consisted of a circular wooden foraging grid (40 cm diameter × 4.5 cm depth) with 8 wells drilled into it that were arranged equidistant from each other around the outside of the grid (Fig. 1d). Wells were rubbed with mozzarella cheese prior to testing to control for olfactory cues and covered with light grey PVC plastic lids. The test consisted of four trials over four consecutive days. For each trial and each individual, one well was randomly chosen to be the rewarded well, with a different well chosen for each trial to ensure there was no confounding effect of memory on performance. To begin each trial, the experimenter baited the rewarded well in view of the focal bird, while the focal bird was attentive (oriented toward the task and looking at the experimenter sensu Scheid and Bugnyar 2008) and within 3 m of the task. The experimenter then stepped back, allowing the focal bird to immediately approach the task and search wells until they located the rewarded well and ate the food reward. This was repeated 24, 48 and 72 h later, with a new well baited and searched by the focal bird, and the observational spatial memory score of an individual was quantified as the cumulative number of wells searched in the four trials prior to locating the rewarded well (including revisits to previously searched wells), with a higher score indicative of worse performance in the task.

### Proxies of motivation and environmental conditions

Individuals, particularly in the wild, may vary in their performance on cognitive tasks depending on a variety of motivational factors including hunger levels or neophobia (Shaw and Schmelz 2017; Boogert et al. 2018). Therefore, for most individuals and tasks, we measured several proxies of motivation: latency to approach the task, body mass, foraging effort, and foraging efficiency. Latency to approach the task, commonly used as a measure of neophobia, was measured as the time taken from when an individual first came within 5 m of the task to when they first touched the task (Ashton et al. 2018b; Soravia et al. 2022). Magpies in the study population have been trained to jump onto an electric top-pan scale for a food reward, allowing for measurement of an individual’s body mass (Ashton et al. 2018a; Pike et al. 2019). All body mass measurements included in analyses were taken within one week of testing. We also noted the order in which birds were tested on each task within each group (test order), to determine if there was any potential confounding effect of social learning or local enhancement on cognitive performance.

We collected ten-minute behavioural focals for each individual (collected on the morning of testing, within four hours of cognitive testing), to investigate whether foraging behaviour correlated with cognitive performance in any task. During behavioural focals, the behaviour of focal individuals was continuously recorded using the CyberTracker programme (CyberTracker Conservation 2021) on an android phone. From behavioural focals, we calculated foraging effort (time spent foraging out of the total focal time), and foraging efficiency (grams of biomass consumed per foraging minute; calculated following Edwards et al. 2015).

Individuals may also differ in their cognitive performance depending on environmental conditions such as temperature (Blackburn et al. 2022; Soravia et al. 2023). Therefore, for each cognitive test conducted, we noted weather condition (cloudy/clear), and obtained temperature measurements from the Bureau of Meteorology records from the nearest weather station, 4 km away (Australian Government Bureau of Meteorology 2023). All cognitive testing was conducted when temperatures were below 27 °C, to ensure no confounding effects of heat stress on cognitive performance (Danner et al. 2021; Blackburn et al. 2022; Soravia et al. 2023).

### Statistical analysis

All statistical analyses were conducted in R studio 4.2.0 (RStudio Team 2022). To ensure magpies were using their memory and not randomly selecting wells in the spatial memory and observational spatial memory tasks, we compared scores to a random search expectation of 4.5 wells (derived from an equation developed from a hypergeometric distribution by Tillé et al. (1996) sensu Ashton et al. 2018a and Shaw et al. 2015). We used non-parametric two-tailed, one-sample Wilcoxon tests to compare the number of lids magpies flipped in each trial to the random search expectation of 4.5 lids.

To investigate whether cognitive performance was affected by proxies of motivation or other factors (i.e. temperature or weather), we performed model selection using generalised linear mixed models (GLMMs) (using the *lme4* package in R (Bates et al. 2015)) on subsets of individuals for whom we had body mass measurements and foraging focals. GLMMs were fitted with a Poisson distribution (with a log link function) and group identity was included as a random factor. We fitted models with latency to approach, body mass, sex, average temperature over testing period, weather conditions (clear/cloudy), test order, foraging effort and foraging efficiency as candidate explanatory terms for performance in each cognitive task. We also included colour and colour shade as an explanatory term for performance in the associative learning task, to investigate whether this was influencing task performance. Model selection was performed using Akaike information criterion values corrected for small sample size (AICc) to test the relative importance of explanatory terms. Models were compared to an intercept only model containing only the intercept and random terms, and only models within two ∆AICc of the best model, and whose predictors had 95% confidence intervals that did not intersect zero, were considered to explain variation in cognitive performance.

To investigate whether cognitive performance was correlated across tasks, we performed Kendall’s rank correlations between the cognitive scores of each pair of tasks and applied a Bonferroni correction to the α level of significance to correct for multiple comparisons. We then performed an unrotated PCA on cognitive scores across the four tasks, using the *FacroMineR* package, to determine if individual performance across tasks could be explained by a single general cognitive factor. Only principal components with an eigenvalue > 1 were considered significant, as per Hopkins et al. (2014) and Shaw et al. (2015). Following Shaw et al. (2015), we then compared the mean and standard deviation for factor loadings on our first principal component, to those obtained in 10,000 random simulations (using the *randomizeMatrix* function in the *picante* package) to determine whether the results from our PCA deviated from random. For each simulation, cognitive scores were randomised between individuals and a PCA was performed, from which the mean and standard deviation of the factor loadings of the first component was extracted. The stored means and standard deviations from the 10,000 simulations were then used to calculate the 95% confidence intervals of the mean and standard deviation, to compare to the mean and standard deviation of the first principal component from our PCA.

## Results

### Individual variation in task performance

Magpies completed the associative learning, spatial memory, and observational spatial memory tasks in an average of 17.47 trials (SE = 1.04, range 10–41, median = 15, *N* = 58), 12.79 trials (SE = 0.85, range 3–25, median = 12, *N* = 57), and 13.79 trials (SE = 0.71, range 4–27, median = 13, *N* = 57) respectively (for all cognitive scores, higher scores indicate worse performance). Magpies in the numerical assessment task chose the larger quantity of food on average 29.65 times (SE = 0.68, range 21–41, median = 29, *N* = 49) out of a total of 45 trials, suggesting magpies are capable of numerical assessment as this ratio represents a significant deviation from random binomial probability (30/45 choices for the larger quantity of food; binomial test: *P* = 0.036; see Supplementary Material Table S1 for information on performance across ratios).

In the spatial memory task, magpies did not search wells significantly differently from random (4.5 wells) in the 24-h trial (mean ± SE = 4.09 ± 0.46, median = 2; one-sample Wilcoxon test: V = 649.5, *P* = 0.16), or the 48-h trial (mean ± SE = 5.02 ± 0.48, median = 5; one-sample Wilcoxon test: V = 876.5, *P* = 0.69), however, in the 72-h trial, magpies did search significantly fewer wells than would be expected if searching randomly (mean ± SE = 3.60 ± 0.35, median = 3; one-sample Wilcoxon test: V = 457, *P* < 0.01), suggesting magpies are able to remember the location of the rewarded well at the 72-h timepoint. The number of wells searched in the olfactory probe trial (mean ± SE = 3.81 ± 0.34, median = 3) was not significantly different from the number of wells searched in the 72-h trial (mean ± SE = 3.60 ± 0.35, median = 3; two-sample Wilcoxon test: V = 253.5, *P* = 0.31), suggesting magpies did not use olfactory cues to locate food rewards.

In the observational spatial memory task, magpies performed significantly better than random in the first trial (mean ± SE = 3.07 ± 0.28, median = 3; one-sample Wilcoxon test: V = 255, *P* < 0.01), second trial (mean ± SE = 3.33 ± 0.32, median = 3; one-sample Wilcoxon test: V = 388.5, *P* < 0.01), third trial (mean ± SE = 4.02 ± 0.33, median = 4; one-sample Wilcoxon test: V = 579.5, *P* < 0.05), and fourth trial (mean ± SE = 3.33 ± 0.33, median = 3; one-sample Wilcoxon test: V = 401, *P* < 0.01), suggesting magpies are able to locate a food reward by observing where an experimenter has put it.

### Proxies of motivation

Cognitive performance was not predicted by any measured proxy of motivation (body mass, latency to approach, foraging efficiency, foraging effort), environmental factors (weather and temperature), test order, or sex (Supplementary Materials Table S2–S5) in any of the tasks.

### Relationship between individual performance across tasks

Individual performance in cognitive tasks was positively related in nearly all (5 out of 6) pairwise comparisons, with all physical tasks being positively correlated with each other, however none of these correlations were statistically significant (Table 1).

**Table 1:** Kendall rank correlation matrix of performance in all cognitive tasks

	Spatial Memory	Observational spatial memory	Associative learning
*Observational spatial memory*			
rs P N	0.083 0.390 56		
*Associative learning*			
rs P N	0.122 0.211 55	0.077 0.433 55	
*Numerical assessment*			
rs P N	0.176 0.140 37	− 0.060 0.616 37	0.128 0.265 41

**Fig. 2:**
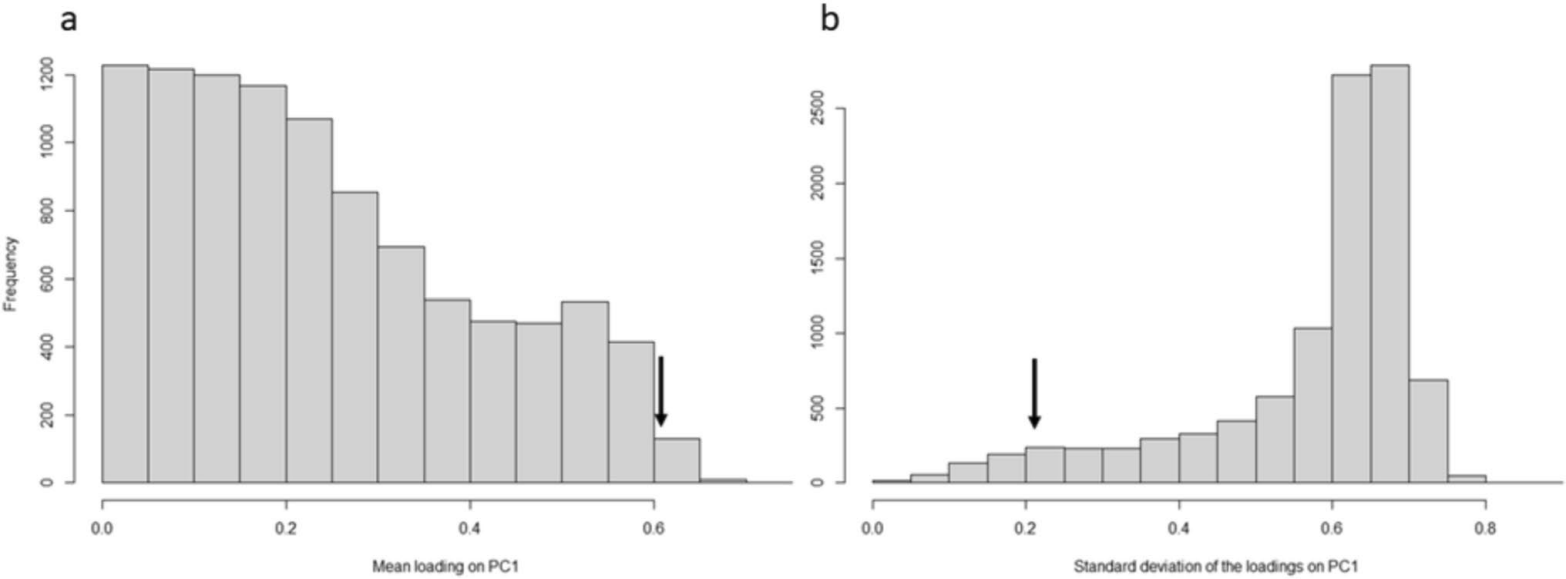
Histogram of **a** the mean and **b** the standard deviations of the cognitive task loadings onto PC1 generated from 10,000 PCA simulations on cognitive scores randomized among individuals. Arrows indicate the observed **a** mean and **b** standard deviation for the first component loadings from our data

A PCA conducted on the performance of 36 individuals who completed all cognitive tasks extracted two components with an eigenvalue > 1 (Table 2). Performance across all tasks loaded positively onto the first component (PC1), though observational spatial memory only loaded weakly (component loading < 0.5) onto PC1 (Table 2). PC1 explained 41.38% of total variation in cognitive performance across tasks (Table 2). Observational spatial memory performance loaded strongly and positively onto a second component (PC2) that also had an eigenvalue > 1 (Table 2). The outcome of 10,000 random simulations revealed that results from the PCA were highly unlikely to have occurred by chance, as the real mean loading onto PC1 was higher than the 95% confidence interval (CI) of the randomly simulated mean loadings (95% CI of simulated means for PC1 = 0.01–0.58, real mean = 0.61; Fig. 2), and the real standard deviation (s.d.) was on the lower end of the distribution of the 95% CI of the simulated s.d. (95% CI of simulated s.d. for PC1 = 0.17–0.72, real s.d. = 0.22; Fig. 2).

**Table 2:** Results of PCA for individuals that completed all 4 cognitive tasks (*N* = 36)

Task	PC1	PC2
Observational spatial memory	0.290	**0.902**
Spatial memory	**0.747**	− 0.087
Associative learning	**0.785**	0.096
Numerical assessment	**0.629**	− 0.432
Eigenvalue	1.655	1.017
% total variance explained	41.38%	25.42%

Loadings and percentage of total variance explained for each principal component with an eigenvalue > 1 are shown. Loadings > 0.5 are in bold

## Discussion

We quantified individual cognitive performance across four tasks (three physical and one social) to investigate whether cognition in Western Australian magpies is underpinned by a general cognitive factor, akin to *g*, or if multiple cognitive domains exist. We found positive pair-wise correlations between all physical cognitive tasks. While none of these pair-wise correlations were significant, positive covariation is suggestive of a common source of variation underlying these tasks, and is often found in studies that report evidence for *g* (Matzel et al. 2003; Keagy et al. 2011; Isden et al. 2013; Shaw et al. 2015). Our PCA revealed that all physical tasks (spatial memory, associative learning, and numerical assessment), but not the social task (observational spatial memory), loaded positively and strongly (component loadings > 0.5) onto the first principal component, with an eigenvalue > 1, and explained 41.38% of the total variation in cognitive performance. A principal component explaining 41.38% of variation in cognitive performance is similar to that reported as *g* in many species, including humans (40%: Plomin and Spinath 2002), laboratory mice (41%: Galsworthy et al. 2005), male spotted bowerbirds (*Ptilonorhynchus maculatus*) (44%: Isden et al. 2013), and New Zealand robins (*Petroica longipes*) (34%: Shaw et al. 2015). Previous work on Western Australian magpies identified a general intelligence factor explaining 64.6% of variation in cognitive performance (Ashton et al. 2018a), however that study, like most other studies on *g* in non-primate species (Galsworthy et al. 2005; Boogert et al. 2011; Isden et al. 2013; Shaw et al. 2015; Shaw and Schmelz 2017), did not include any tasks assessing social cognition.

The PCA also produced a second principal component with an eigenvalue > 1, onto which our social cognitive task (observational spatial memory) loaded strongly and positively (component loading of 0.902), while all other (physical) tasks loaded weakly (component loadings < 0.5), and were therefore not considered salient (*per* Hopkins et al. 2014). In addition, the weakest correlations seen between pairs of tasks in this study were between the social task and each of the three physical tasks. These findings reveal that performance on the social task, observational spatial memory, does not align with performance on the physical tasks, and suggests that some aspect of this task separates it from the physical cognitive tasks. Cognition in this species therefore does not appear to be underpinned by one general cognitive factor. Although less common than studies supporting the existence of *g* in non-primate animal species (potentially due to limited studies that include social tasks), some studies have found support for the existence of multiple cognitive domains. For example, a battery of six cognitive measures and two non-cognitive measures (stress and activity) administered to 60 captive mice identified four principal components with eigenvalues greater than one (Locurto et al. 2003). While one of these components appeared to mark stress only (one of the two non-cognitive measures included in the study), two cognitive tasks loaded onto each of the other three components, suggesting the cognitive tasks measured in this study comprised three distinct domains. These three cognitive components could not be clearly defined however, due to a relatively low number of tasks compared to the large number of extracted components (Locurto et al. 2003). Similarly, in wild satin bowerbirds (*Ptilonoryhnchus violaceus*), a PCA conducted across six tasks (two problem-solving tasks, three bower rebuilding tasks, and a measure of mimetic repertoire size) identified three principal components with eigenvalues greater than one (Keagy et al. 2011). However, it was unclear what cognitive abilities these tasks and therefore domains represented (Shaw and Schmelz 2017), and previous work has criticised the use of problem-solving tasks as a robust measure of cognition (Thornton et al. 2014; van Horik and Madden 2016).

Performance on the four cognitive tasks used in this study was not affected by any of our measures of motivation (foraging effort, foraging efficiency, or body mass), suggesting motivation was unlikely to be confounding task performance. This is supported by the fact that all birds completed all the cognitive tasks that they were tested on. Latency to approach the task also had no effect on the performance of individuals, suggesting bolder individuals in this species do not outperform shyer individuals. This is supported by previous work on this population that found no effect of trapability or self-selection on cognitive performance (Ashton et al. 2022b).

In contrast to previous work on this study species using a spatial memory task that tested birds only 24 and 48 h after the training period (i.e. no 72-h trial) and found that magpies searched significantly better than random at the 48 h trial (Ashton et al. 2018a), we found that magpies searched randomly at the 24 and 48-h spatial memory trials, but not the 72-h trial. A recent study that quantified long term repeatability in this species found that spatial memory performance was not repeatable in the long term (Ashton et al. 2022a), which may explain why results from our 2023 spatial memory testing differed from results from testing between 2013 and 2015 (Ashton et al. 2018a). In addition, when considering the sum of only the 24- and 48- hour spatial memory trials, the mean and range of scores achieved by birds in the present study is not dissimilar from the mean and range of scores reported in 2015 (present study 24 h and 48 h score: mean = 8.61 ± 0.63, range 2–22; 2015 24 h and 48 h score (Ashton et al. 2022a): mean = 8.53 ± 0.99, range 2–27). That the spatial memory performance of magpies improved over trials is a common finding in tasks assessing spatial memory in animals (Sewall et al. 2013; Ashton et al. 2018a; MacKinlay and Shaw 2019; Shaw et al. 2019), and suggests that repeated trials help individuals to consolidate and better remember the location of the rewarded well.

For our social cognition test, a potential confound was the use of a human demonstrator, rather than a conspecific, which may not be an ecologically relevant test of magpie responses to “social cues” (Shaw and Schmelz 2017). However, the use of a human demonstrator in such experiments is common (Herrmann et al. 2007; Scheid and Bugnyar 2008; Pika et al. 2020), and the basic cognitive requirements of learning and inferring the location of food from a heterospecific is likely to be similar to learning from a conspecific. In our task measuring numerical assessment, we could not exclude the possibility that birds may have used other cues (such as the volume or area taken up by the food reward) when selecting a board. This is a potential confound in many studies assessing numerical assessment (Simona et al. 2022; Khatiwada and Burmeister 2022; Tomonaga et al. 2023), and future studies should utilise experiments presenting differing numerical ratios with the same total area or volume, as well as presenting only the numerical cues and not food rewards to individuals when making their choice.

Here, we aimed to identify if performance in a social cognition task correlated with performance in physical cognition tasks that are commonly tested in wild birds. Our results provide tentative support for the existence of two separate cognitive domains in Western Australian magpies and highlights the importance of including tasks measuring social cognition in test batteries presented to animals. Future studies should aim to utilise a more comprehensive battery of cognitive tasks compared to that which has been used in this and previous studies in wild birds (Isden et al. 2013; Shaw et al. 2015; Ashton et al. 2018a; Boogert et al. 2018; Soravia et al. 2022), that spans *both* social and physical cognitive tasks. As the present study only included one social cognitive task, we cannot comprehensively conclude whether it was the social aspect of this task that separated it from the physical tasks, and therefore future studies on birds should prioritise the inclusion of multiple distinct tasks assessing social cognition. Presenting multiple social and physical cognitive tasks to birds in the wild will enable a more comprehensive and conclusive investigation of whether social and physical cognition align.

## Supplementary Information

Below is the link to the electronic supplementary material.

Supplementary file1 (DOCX 1463 KB)

## Data Availability

Data accompanying this article can be found on figshare at https://figshare.com/s/f6d1055c12a280f0bcfa.
