# Supplementary material for: Investigating the relationship between physical cognitive tasks and a social cognitive task in a wild bird
Source: Anim Cogn. 2024 Jul 26;27(1):52. doi: 10.1007/s10071-024-01892-4 (PMC11281958; doi:10.1007/s10071-024-01892-4)
Supplement: Supplementary file 1 — Supplementary file1 (DOCX 1463 KB) [file 10071_2024_1892_MOESM1_ESM.docx]

***Investigating the relationship between physical cognitive tasks and a social cognitive task in a wild bird***

**Supplementary material**


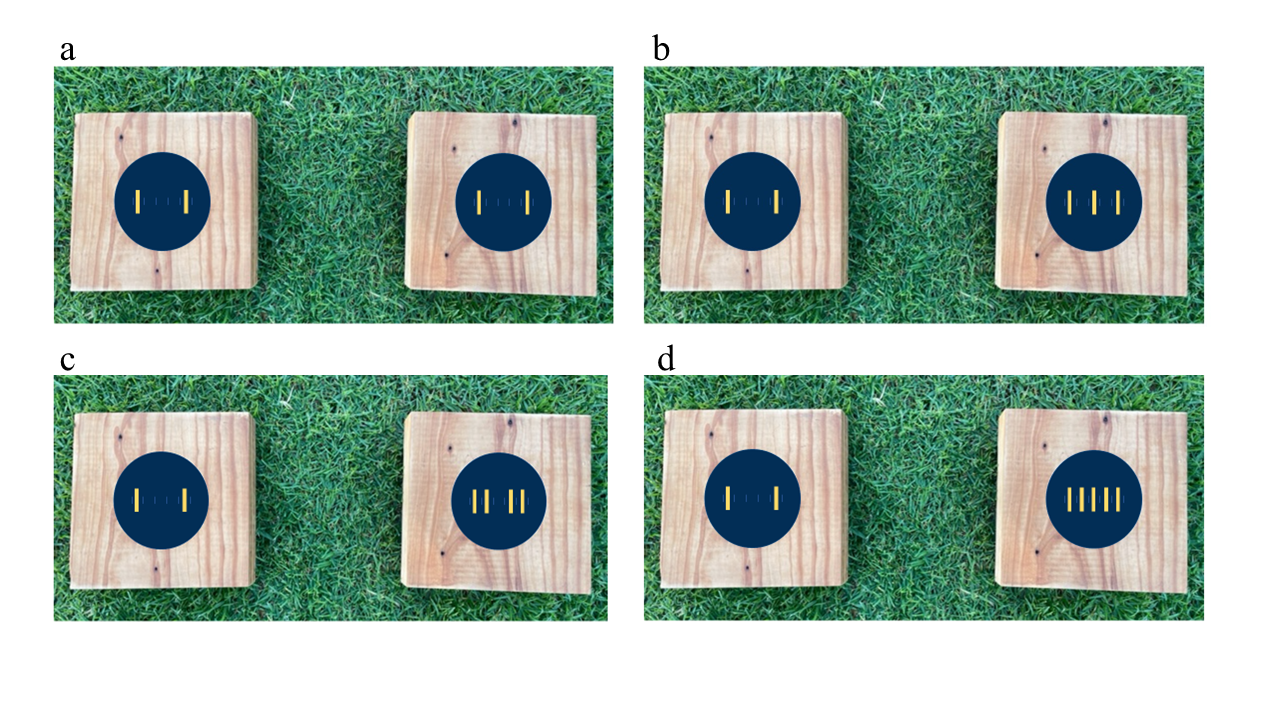


**Figure S1.** Numerical assessment task: a) 2 v 2 ratio, b) 2 v 3 ratio, c) 2 v 4 ratio, d) 2 v 5 ratio.

**Table S1.** Average numerical assessment scores on each ratio presented. Higher score is indicative of worse performance. *N* = 49 birds.

|  | **2 v 3** | **2 v 4** | **2 v 5** |
| --- | --- | --- | --- |
| **Mean (±SE) number of times smaller quantity was chosen (out of 15 trials)** | 6.12 (± 0.27) | 5.14 (± 0.33) | 4.08 (± 0.37) |

**Table S2.** Full model selection output for candidate terms affecting spatial memory performance on a subset of individuals with foraging focals and body mass measurements. All models included group ID as a random term. Corrected Akaike information criterion (AICc) and ∆AICc are provided for each candidate model. Only within 2 AICc of the top model, and with predictors whose 95% confidence intervals did not intersect zero were included in the top model set and are highlighted in bold. *N =* 44 birds from 15 groups.

| **Predictor** | **AICc** | **∆AICc** |
| --- | --- | --- |
| **Basic (intercept only)** | **327.19** | **0.00** |
| Latency to approach^1^ | 327.59 | 0.41 |
| Sex^1^ | 328.13 | 0.95 |
| Average temperature^1^ | 328.79 | 1.60 |
| Weather^1^ | 328.96 | 1.77 |
| Foraging efficiency^1^ | 329.04 | 1.86 |
| Test order | 329.19 | 2.00 |
| Foraging effort | 329.45 | 2.27 |
| Body mass | 329.46 | 2.27 |

^1^Although within 2 AICc of the top model, not included in top model set as it is within 2AICc of a simpler model.

**Table S3.** Full model selection output for candidate terms affecting observational spatial memory performance on a subset of individuals with foraging focals and body mass measurements. All models included group ID as a random term. Corrected Akaike information criterion (AICc) and ∆AICc are provided for each candidate model. Only within 2 AICc of the top model, and with predictors whose 95% confidence intervals did not intersect zero were included in the top model set and are highlighted in bold. *N =* 45 birds from 14 groups.

| **Predictor** | **AICc** | **∆AICc** |
| --- | --- | --- |
| Average temperature^1^ | 277.77 | 0.00 |
| **Basic (intercept only)** | **278.80** | **1.03** |
| Body mass^1^ | 279.43 | 1.66 |
| Foraging efficiency | 279.92 | 2.15 |
| Latency to approach | 280.34 | 2.57 |
| Foraging effort | 280.57 | 2.80 |
| Weather | 280.90 | 3.13 |
| Test order | 281.03 | 3.26 |
| Sex | 281.06 | 3.28 |

^1^Although within 2 AICc of the top model, not included in top model set as it is within 2AICc of a simpler model.

**Table S4.** Full model selection output for candidate terms affecting numerical assessment performance on a subset of individuals with foraging focals and body mass measurements. All models included group ID as a random term. Corrected Akaike information criterion (AICc) and ∆AICc are provided for each candidate model. Only within 2 AICc of the top model, and with predictors whose 95% confidence intervals did not intersect zero were included in the top model set and are highlighted in bold. *N =* 23 birds from 8 groups.

| **Predictor** | **AICc** | **∆AICc** |
| --- | --- | --- |
| **Basic (intercept only)** | 130.60 | 0.00 |
| Test order^1^ | 130.75 | 0.15 |
| Foraging efficiency^1^ | 131.26 | 0.66 |
| Foraging effort^1^ | 132.55 | 1.95 |
| Latency to approach^1^ | 132.58 | 1.98 |
| Body mass | 132.72 | 2.12 |
| Average temperature | 133.17 | 2.57 |
| Weather | 133.17 | 2.57 |
| Sex | 133.21 | 2.61 |

^1^Although within 2 AICc of the top model, not included in top model set as it is within 2AICc of a simpler model.

**Table S5.** Full model selection output for candidate terms affecting associative learning performance on a subset of individuals with foraging focals and body mass measurements. All models included group ID as a random term. Corrected Akaike information criterion (AICc) and ∆AICc are provided for each candidate model. Only within 2 AICc of the top model, and with predictors whose 95% confidence intervals did not intersect zero were included in the top model set and are highlighted in bold. *N =* 17 birds from 13 groups.

| **Predictor** | **AICc** | **∆AICc** |
| --- | --- | --- |
| **Basic (intercept only)** | 119.66 | 0.00 |
| Foraging efficiency^1^ | 120.26 | 0.60 |
| Sex | 121.69 | 2.03 |
| Body mass | 121.76 | 2.10 |
| Latency to approach | 121.82 | 2.16 |
| Test order | 121.91 | 2.25 |
| Foraging effort | 122.04 | 2.37 |
| Weather | 122.05 | 2.39 |
| Average temperature | 122.62 | 2.96 |
| Colour | 129.76 | 10.10 |

^1^Although within 2 AICc of the top model, not included in top model set as it is within 2AICc of a simpler model.
